# Cognitive and Surgical Outcome in Mesial Temporal Lobe Epilepsy Associated with Hippocampal Sclerosis Plus Neurocysticercosis: A Cohort Study

**DOI:** 10.1371/journal.pone.0060949

**Published:** 2013-04-22

**Authors:** Marino M. Bianchin, Tonicarlo R. Velasco, Erica R. Coimbra, Ana C. Gargaro, Sara R. Escorsi-Rosset, Lauro Wichert-Ana, Vera C. Terra, Veriano Alexandre, David Araujo, Antonio Carlos dos Santos, Regina M. F. Fernandes, João A. Assirati, Carlos G. Carlotti, João P. Leite, Osvaldo M. Takayanagui, Hans J. Markowitsch, Américo C. Sakamoto

**Affiliations:** 1 Center for Epilepsy Surgery (CIREP), Hospital das Clínicas de Ribeirão Preto, Department of Neurology, Ribeirão Preto School of Medicine, University of São Paulo, Ribeirão Preto, São Paulo, Brazil; 2 Basic Research and Advanced Investigations in Neurology (B.R.A.I.N.), Division of Neurology, Hospital de Clínicas de Porto Alegre, Rio Grande do Sul, UFRGS, Brazil; 3 Department of Radiology, Ribeirão Preto School of Medicine, University of São Paulo, Ribeirão Preto, São Paulo, Brazil; 4 Department of Neurosciences and Behavior, Ribeirão Preto School of Medicine, University of São Paulo, Ribeirão Preto, São Paulo, Brazil; 5 Neurosurgery Division, Department of Surgery and Anatomy, Ribeirão Preto School of Medicine, University of São Paulo at Ribeirão Preto, São Paulo, Brazil; 6 Arbeitseinheit Physiologische Psychologie, Universität Bielefeld, Bielefeld, Germany; Emory University, Georgia Institute of Technology, United States of America

## Abstract

**Background:**

Where neurocysticercosis (NCC) is endemic, chronic calcified neurocysticercosis (cNCC) can be observed in patients with mesial temporal lobe epilepsy associated with hippocampal sclerosis (MTLE-HS). Considering that both disorders cause recurrent seizures or cognitive impairment, we evaluated if temporal lobectomy is cognitively safe and effective for seizure control in MTLE-HS plus cNCC.

**Methods:**

Retrospective cohort study of neuropsychological profile and surgical outcome of 324 MTLE-HS patients submitted to temporal lobectomy, comparing the results according to the presence or absence of cNCC.

**Findings:**

cNCC occurred in 126 (38.9%) of our MTLE-HS patients, a frequency higher than expected, more frequently in women than in men (O.R. = 1.66; 95% C.I. = 1.05–2.61; *p* = 0.03). Left-side (but not right side) surgery caused impairment in selected neuropsychological tests, but this impairment was not accentuated by the presence of cNCC. Ninety-four (74.6%) patients with MTLE-HS plus cNCC and 153 patients (77.3%) with MTLE-HS alone were Engel class I after surgery (O.R. = 1.16; 95% C.I. = 0.69–1.95; *p* = 0.58). However, the chances of Engel class IA were significantly lower in MTLE-HS plus cNCC than in patients with MTLE-HS alone (31.7% versus 48.5%; O.R. = 2.02; 95% C.I. = 1.27–3.23; *p* = 0.003). Patients with MTLE-HS plus cNCC showed higher rates of Engel class ID (15.1% versus 6.6%; O.R. = 2.50; 95% C.I. = 1.20–5.32; *p* = 0.012).

**Interpretation:**

cNCC can be highly prevalent among MTLE-HS patients living in areas where neurocysticercosis is endemic, suggesting a cause-effect relationship between the two diseases. cNCC does not add further risk for cognitive decline after surgery in MTLE-HS patients. The rates of Engel class I outcome were very similar for the two groups; however, MTLE-HS plus cNCC patients achieved Engel IA status less frequently, and Engel ID status more frequently. Temporal lobectomy can be safely performed in most patients with MTLE-HS plus cNCC without affecting cognitive outcome. Long-term surgical seizure control in MTLE-HS plus cNCC is still satisfactory, as long as selected patients remain under medication.

## Introduction

Epilepsy is a common disorder affecting at least 0.5% of the population in developed countries. It is even more frequent in developing countries where most of all epileptic patients are living [1]–[3]. Mesial temporal lobe epilepsy associated with hippocampal sclerosis (MTLE-HS) is one of the most common forms of focal epilepsy. It is frequently resistant to antiepileptic drugs, and patients might benefit from surgery [4]–[6]. One of the major concerns of temporal lobectomy for MTLE-HS treatment is that patients might experience cognitive decline after the procedure [7]–[10]. In this respect, seizure control is important for cognitive outcome after surgery [7]. Thus, an important aspect of presurgical evaluation of mesial temporal lobectomy is the identification of patients at higher risk of poor seizure control as well as those at risk for cognitive decline after temporal lobectomy.

**Figure 1:**
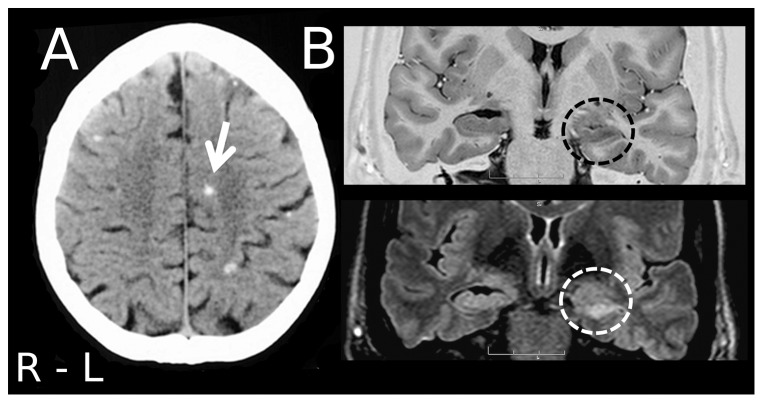
Neuroimaging of a patient with epilepsy with both, neurocysticercosis and hippocampal sclerosis. (A) A CT-scan shows calcified lesions typical of neurocysticercosis (arrow). (B) MRI of this patient shows abnormalities typical of mesial temporal lobe epilepsy associated with hippocampal sclerosis (circles).

Neurocysticercosis (NCC) is the world's most common neurological infectious disease. The pathology is endemic in Latin America, India, Asia, and Africa. In developed countries the disease is less common but still a public health problem in some regions [10]–[14]. NCC is usually associated with many neurological complaints, including seizures, cognitive deficits, depression, apathy, emotional instability, and hallucinations [14]–[22]. Because of the elevated prevalence of NCC in endemic regions, it is not uncommon to observe patients with calcified chronic neurocysticercosis (cNCC) associated with hippocampal sclerosis [22]–[24]. Considering that both disorders are potentially epileptogenic and associated with cognitive symptoms, the spectrum of MTLE-HS plus cNCC might lead to considerable diagnostic confusion and prognostic concerns, especially when patients require surgical treatment (Figure 1).

Cognitive reserve can be seen as the capacity of the mature adult brain to sustain the effects of disease or injury sufficient to cause cognitive impairment in an individual possessing less cognitive reserve [25]. Although several cognitive disorders have been observed in MTLE-HS patients or in NCC patients, the cognitive repercussions of cNCC associated with MTLE-HS or the impact of surgical treatment in patients suffering from both disorders have been poorly studied. Indeed, because of the concept of cognitive reserve, additional damage to cognitive neural networks already damaged may lead to additional unpredictable cognitive impairments. We have previously observed that patients with cNCC plus MTLE-HS can become seizure free after temporal lobectomy when seizures are proved to originate from the mesial temporal lobe region [23], [24]. However, this conclusion was based on few patients and, although valid for generating a hypothesis, it might not be sufficient to establish medical recommendations. Moreover, concern also arises regarding the degree of cognitive decline after temporal lobectomy in patients with MTLE-HS plus cNCC. Because of insufficient studies it has not been established whether or not cNCC is a risk factor for poor seizure control or cognitive decline after temporal lobectomy in patients with MTLE-HS associated with cNCC. These unsolved matters deserve further investigation.

In the present study we analyzed a large cohort of patients with MTLE-HS from an area where NCC is endemic, regarding their pre- and postsurgical cognitive performance after temporal lobectomy for treatment of MTLE-HS. The patients were also evaluated also evaluated in terms of surgical seizure control. This study is important because it might support surgical decisions and treatments for patients with MTLE-HS plus cNCC, an association that is becoming increasingly recognized and that in fact might be quite common in several world regions where NCC is still endemic.

## Materials and Methods

### Ethics statement

The study was approved the Research Ethics Committee of the University Hospital, Faculty of Medicine of Ribeirão Preto, University of são Paulo, and was conducted according to the principles expressed in the Declaration of Helsinki. All patients gave written informed consent to participate.

### Study population

We retrospectively studied a cohort of 350 consecutive patients who were surgically treated for MTLE-HS at CIREP, Center for Epilepsy Surgery at Ribeirão Preto, from 1995 to 2004. Twenty-six patients were excluded due to impossibility to review all neuroimaging data or surgical outcome at the time of the study. The data of the remaining 324 patients were analyzed. The inclusion criteria were: 1) seizure semiology consistent with MTLE, usually with epigastric, autonomic or psychic auras, followed by behavioral arrest, progressive clouding of consciousness, oroalimentary and manual automatisms, and autonomic phenomena; 2) anterior and mesial temporal interictal spikes; 3) no lesion other than calcification compatible with cNCC in CT-scan or MRI (Figure 1) and atrophy and increased signal in the hippocampal formation identified by MRI (Figure 1); 4) histopathological examination compatible with hippocampal sclerosis (HS); 6) presurgical investigation compatible with unilateral seizure onset. The exclusion criteria were: 1) focal neurological abnormalities on physical examination; 2) generalized or extratemporal EEG spikes; 3) marked cognitive impairment on neuropsychological testing; 4) neuroimaging findings other than mesial temporal lobe sclerosis or cNCC.

During the preoperative period, patients were submitted to a uniform presurgical evaluation that included detailed neuropsychological evaluation. Six to nine months after surgery, the patients were re-submitted to the same battery of neuropsychological tests in order to assess the cognitive repercussions of temporal lobectomy. The pharmacological treatment of each patient was optimized before surgical treatment and maintained after the procedure and neuropsychological evaluation. Thus, after surgery, during the postsurgical test session, pharmacological treatment was left unchanged compared to the preoperative evaluation. Our study is in accordance with the STROBE requirements.

### Diagnosis of Neurocysticercosis

Definitive neurocysticercosis was diagnosed if the following criteria were met (i) an absolute criterion, such as histological demonstration of the parasite or cystic lesions showing the scolex on CT or MRI; (ii) two major criteria, such as lesions highly suggestive of neurocysticercosis on neuroimaging studies, spontaneously resolving small single enhancing lesions, or resolution of intracranial cystic lesions after therapy with albendazole or praziquantel; or (iii) one major and two minor criteria, such as lesions compatible with neurocysticercosis on neuroimaging studies, clinical manifestations suggestive of neurocysticercosis, and positive CSF ELISA for the detection of anticysticercal antibodies, plus epidemiological evidence. According to the above criteria, the presence of solid, dense, supratentorial calcifications, 1–10 mm in diameter, in the absence of other illnesses should be considered to be highly suggestive of neurocysticercosis (Figure 1) [26], [27].

### Neuroimaging

All neuroimaging findings were carefully and independently reviewed by neuroradiologists skilled in epilepsy or in NCC neuroimaging (ACdS, DA Jr., LWA). The calcified lesions were computed and classified according to the lobe in which they were observed and are presented in Table 1. For analysis, subjects were divided into patients with MTLE-HS plus cNCC (HS-NCC group) (*n* = 126) and patients with MTLE-HS alone (HS-only group) (*n* = 198), according to neuroimaging findings.

**Table 1:** Distribution of cysticerci according to cerebral region.

	Total	Minimum	Maximum	Mean	Std. Deviation
Left Frontal	104	0	17	0.87	2.43
Right Frontal	100	0	15	0.79	1.89
Left Temporal	55	0	07	0.46	1.02
Right Temporal	48	0	06	0.41	0.95
Left Parietal	41	0	05	0.34	0.83
Right Parietal	59	0	06	0.49	0.98
Left Occipital	49	0	09	0.41	1.09
Right Occipital	55	0	08	0.59	1.06
Total	511	0	17	-	-

### Clinical parameters and presurgical evaluation of patients

Clinical characteristics included sex, age at surgery, age at epilepsy onset (recurrent seizures), duration of epilepsy, number of seizures/month, years of education, occupational history, side of surgery, MRI findings, lateralization of interictal spikes, ictal findings, pharmacotherapy, and surgical outcome according to Engel's classification.

Presurgical evaluation was performed by an experienced multidisciplinary team and included a detailed clinical history and neurological examination, interictal and ictal video-EEG analysis, structural and functional imaging, psychiatric evaluation, neuropsychological testing, and, when appropriate, the intracarotid amobarbital test (Wada's test) for memory and speech representation. As part of the presurgical evaluation, all patients were submitted to prolonged video-EEG recording (Vangard System, Cleveland, OH, USA) using scalp electrodes and sphenoidal electrodes. Interictal spikes were assessed by visual analysis as previously described and classified as: 1) predominantly unilateral interictal spikes, if 70% or more of all spikes occurred in one temporal lobe, 2) no spikes, if no spikes were observed during video-EEG, or 3) bilateral, in all other cases. At least four seizures were recorded for each patient. In all cases, the epileptogenic zone (EZ) was inferred on the basis of the clinical, neuroimaging, neuropsychological and electrophysiological results. When the EZ could not be estimated noninvasively, patients underwent intracranial EEG recordings.

### Surgical procedure and postoperative follow-up

The surgical approach was similar for all patients. One of two neurosurgeons experienced in surgery for epilepsy (JAA Jr. or CGC Jr.) resected a maximum of 4.0 to 5.0 cm of the anterior lateral temporal lobe. The mesial resection included 2/3 of the amygdala and the anterior 2.0 to 3.0 cm of the hippocampus. All patients had anathomopathological confirmation of mesial temporal sclerosis.

Surgical outcome was defined by seizure status determined during outpatient clinical interviews. For seizure outcome, we used Engels' classification. Seizure outcome was assessed during routine follow-up by experienced epileptologists who were blind to the presurgical parameters or the design of this study. Most patients included in this cohort were available for the evaluation of surgical outcome and continued to be followed up in our service at the end of the present study.

### Neuropsychological evaluation

Patients were submitted to presurgical neuropsychological tests during routine pre-surgical evaluation. Postsurgical neuropsychological evaluation was homogeneous for the two groups of patients and was performed 7.5 months after surgery for the HS-only group and 7.6 months after surgery for the HS-NCC group (*p* = 0.88). For the purpose of the present study we included 19 cognitive tests. To determine intelligence, we evaluated IQ. Memory tests included Logical Memory I, Logical Memory II, Visual Reproduction I, and Visual Reproduction II of the Wechsler Memory Scale-revised. We also analyzed the Rey Auditory Verbal Learning Test (RAVLT), RAVLT retention, RAVLT recognition memory (RAVLT REC), and delayed recall of the RAVLT, Rey Visual Design Learning Test (RVDLT), RVDLT retention, RVDLT recognition memory (RVDLT REC), and delayed recall (30 minutes) of the RVDLT, Copy of Rey-Osterrieth Complex Figure, delayed recall (30 minutes) of the Rey-Osterrieth Complex Figure, Digit Span and Inverted Digit Span (WMS-R), Boston Naming Test, and Word Fluency (FAS). In addition to the IQ test, the premorbid skills of the patients were also indirectly estimated on the basis of their educational level.

### Data Collection and Statistical Analysis

Patients were given a single identification number that was used for all analyses, which were done anonymously. Continuous variables were analyzed by the Student t-test or Mann-Whitney test depending on the normality of data distribution. Categorical variables were analyzed by the Chi-square test. Results of cognitive performance were evaluated by multivariate analysis of variance with repeated measures (MANOVA with repeated measures), with the presence or absence of cNCC as an independent between-group variable and pre- or postsurgical neurocognitive evaluation as a within-subject variable. Years of education is a variable that influences cognitive profile and was included as a covariate. The level of significance in the multivariate test was established using Wilk's Lambda test. Thus, in the present study each patient was considered to be his/her own control and scores of cognitive tests were compared in terms of pre- and postsurgical performance, split by the presence or absence of cNCC, with educational level controlled as a covariate. For the neuropsychological tests, the results are reported as mean ± SD, the level of significance for differences between pre- and postsurgical evaluation (variability of cognitive performance caused by temporal lobectomy), the additional effect of NCC alone, and the interaction of the effect of NCC and of surgery. Effect of educational level was homogeneous and highly significant for all tests (*p<*0.001) and for this reason it is not presented. The results of the more important tests in which we observed a significant difference in neuropsychological performance due to surgery are plotted in SPSS generated graphics in order to facilitate the understanding of the analysis model used. In graphs, the levels of significance are indicated only for surgery effect since there was no significant decline in cognitive performance due to the presence of cNCC. All statistical analyses were performed using the SPSS version 17.0 software (SPSS Inc., Chicago, IL, USA). In cognitive tests, because of multiple correlations we set our level of significance at *p*<0.01 in order to prevent Type I error. Bonferroni adjustments were not used because of excessive comparisons, leading to an unacceptable increase of Type II error [28].

## Results

Considering the complete cohort, 126 (38.9%) of the 324 patients initially selected for this study presented radiological findings compatible with cNCC, a prevalence far higher than expected for the population living in our region [29], [30]. The results for cysticercus localization are presented in Table 1. cNCC was observed to a large extent in the frontal lobes. The results regarding clinical aspects and complementary exams are presented in Table 2. cNCC was more frequently observed in women. Age at surgery was slightly higher in the HS-NCC group. No other differences were observed between HS-NCC and HS-only groups (Table 2). For seizure outcome, the mean postsurgical follow-up time when this study was completed was 67.88 months (range: 41–124 months) and there was no difference between HS-only and HS-NCC regarding follow-up time (*p* = 0.49). Surgical outcome is presented in Table 3. In this Table, outcomes are presented in four different ways according to Engel's classification. Overall, the rates of Engel class I (IA+IB+IC+ID) outcome were closely similar for the two groups (O.R. = 1.16; 95% C.I. = 0.69–1.95; *p* = 0.58), however MTLE-HS plus cNCC patients achieved Engel class IA status less frequently (31.7% versus 48.5%; O.R. = 2.02; 95% C.I. = 1.27–3.23; *p* = 0.003), and Engel class ID status more frequently (15.1% versus 6.6%; O.R. = 2.50; 95% C.I. = 1.20–5.32; *p* = 0.012).

**Table 2:** Pre-surgical parameters of MTLE-HS patients with (HS-NCC) and without (HS-ONLY) chronic calcified neurocysticercosis.

Variable	HS-ONLY *n* = 198 (61.1%)	HS-NCC *n* = 126 (38.9%)	O.R. 95% CI	*p*
Sex				
Male	100 (45.7%)	48 (38.1%)		
Female	98 (54.3%)	78 (61.9%)	1.66 (1.05–2.61)	0.03*
Occupation				
Unskilled labor or inactive	118(59.6%)	47 (37.3%)		
Semi-skilled or skilled labor	80(40.4%)	79 (62.7%)	1.14 (0.72–1.80)	0.58
Age at surgery (y)				
Mean	36.56	38.75		
SD	9.66	8.94	-	0.04*
Age at epilepsy onset (y)				
Mean	9.39	9.82		
SD	8.51	7.95	-	0.65
Disease duration (y)				
Mean	27.17	28.93		
SD	11.23	10.77	-	0.16
Years of education				
Mean	6.75	6.31		
SD	4.22	4.36	-	0.37
Number of seizures/month				
Mean	8.23	8.58		
SD	8.14	8.97	-	0.22
Pharmacotherapy				
Monotherapy	46 (23.2%)	28 (22.2%)		
Polytherapy	152 (76.8%)	98 (77.8%)	1.06 (0.62–1.81)	0.89
Associated benzodiazepines				
No	45 (22.7%)	29 (23.02%)		
Yes	153 (77.3%)	97 (76.98%)	0.98 (0.58–1.67)	1.00
Interictal spikes				
Predom. Unilateral/Normal	158(79.8%)	94 (74.6%)		
Bilateral	40(20.2%)	32 (25.4%)	1.34 (0.79–2.20)	0.28
Ictal EEG				
Unilateral	186(93.9%)	116 (92.1%)		
Bilateral	12( 06.1%)	10 ( 07.9%)	1.34 (0.56–3.10)	0.50
Side of Surgery				
Left	105(53.0%)	69 (54.8%)		
Right	93(47.0%)	57 (45.2%)	1.07 (0.68–1.68)	0.76

(*) significant.

**Table 3:** Surgical outcome according to Engel classification.

Surgical Outcome	HS-ONLY *n* = 198 (61.1%)	HS-NCC *n* = 126 (38.9%)	O.R. 95% CI	*p*
Engel I versus Other (Engel II+III+IV)				
Engel I (IA+ IB+IC+ID)	153 (77.3%)	94 (74.6%)		
Other (Engel II+III+IV)	45 (22.7%)	32 (25.4%)	1.16 (0.69–1.95)	0.58
Engel Subtypes				
Engel IA	96 (48.5%)	40 (31.7%)		
Engel IB	30 (15.2%)	25 (19.8%)		
Engel IC	14 (7.1%)	10 (7.9%)		
Engel ID	13 (6.6%)	19 (15.1%)		
Engel II	17 (8.6%)	08 (6.3%)		
Engel III	21 (10.6%)	16 (12.7%)		
Engel IV	07 (3.5%)	08 (6.3%)	-	0.03*
Engel IA versus Other Engel Subtypes				
Engel IA	96 (48.5%)	40 (31.7%)		
Other (IA+IB+IC+ID+II+III+IV)	102 (51.5%)	86 (68.3%)	2.02 (1.27–3.23)	0.03*
Engel ID versus Other Engel Subtypes				
Engel ID	13 (06.6%)	19 (15.1%)		
Other (IA+IB+IC+II+III+IV)	185 (93.4%)	107 (84.9%)	2.50 (1.20–5.32)	0.012*

(*) significant.

**Figure 2:**
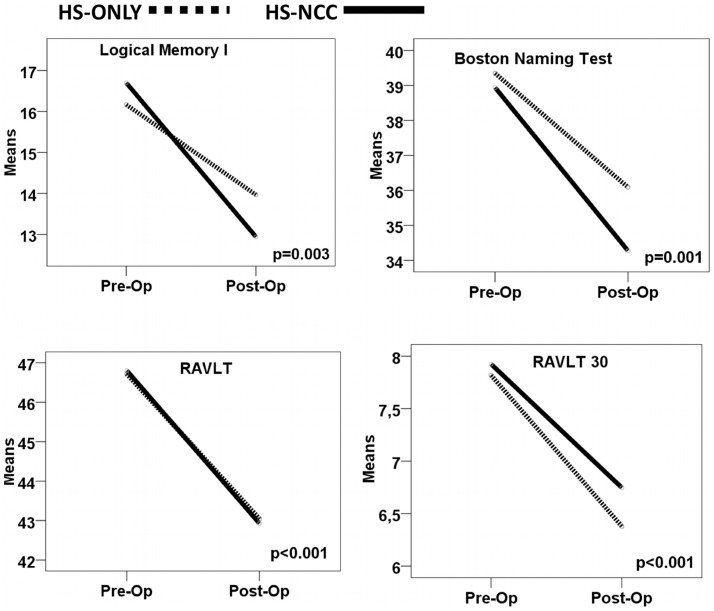
Pre- and post- surgical cognitive performance in selected tests for left temporal lobectomy. Surgery for treatment of left MTLE-HS significantly reduced selected neuropsychological test performance uniformly in both groups (see text). The presence of cNCC did not cause any additional cognitive loss. The continuous line represents HS-NCC group and the dotted line represents the HS-ONLY group.

**Figure 3:**
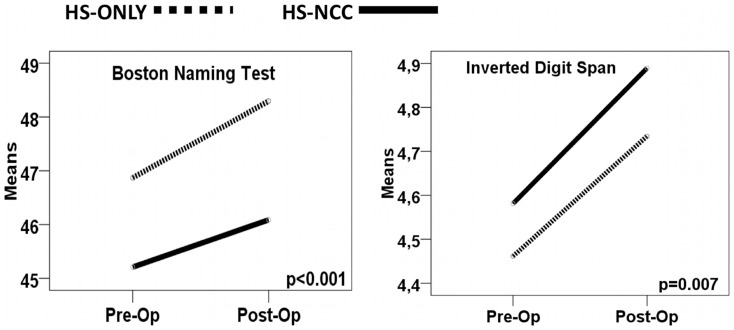
Pre- and post- surgical cognitive performance in selected tests for right temporal lobectomy. Surgery for treatment of right MTLE-HS significantly improved selected neuropsychological test performance uniformly in both groups (see text). The presence of cNCC did not cause any additional cognitive loss. The continuous line represents the HS-NCC group and the dotted line represents the HS-ONLY group.

The neuropsychological evaluations are presented in Table 4 for left side surgery, in Table 5 for right side surgery, and in Figures 2 for left side surgery and Figure 3 for right side surgery. Test-by-test analysis of the neuropsychological results, controlled by educational level, showed that the presence of cNCC did not add a significant burden to neuropsychological decline after surgery in MTLE-HS patients. Both groups of patients had the same average educational level of 6 years, meaning that, on average, they went to school until about 12 years of age (6th grade). The effect of educational level was homogeneous and highly significant for all tests (*p*<0.001). For this reason it is not presented in Tables 4 and 5. As expected, left temporal lobectomy decreased performance in selected neuropsychological tests (Table 4 and Figure 2). However, the presence of cNCC did not cause additional impairment. Regarding right MTLE-HS, right temporal lobectomy did not provoke deficits in neuropsychological tests. Interestingly, after surgery, right MTLE-HS patients showed improvement in the Inverted Digit Span and Boston Naming Test (Table 5 and Figure 3), possibly due to better seizure control.Because of the unexpected gender difference, we also split our sample by gender, carrying out the same analyses (data not showed). This resulted in similar conclusions, demonstrating that cNCC did not add any additional cognitive decline when temporal lobectomy was indicated for treatment of refractory MTLE-HS. Taken together, our results suggest that surgery affected cognitive performance mainly in patients with left MTLE-HS and this effect was similar for HS-NCC and HS-only patients. Thus, we concluded that, when associated with MTLE-HS, cNCC did not lead to any additional cognitive decline.

**Table 4:** Results of Pre- and Postsurgical Neuropsychological Performance for Left Mesial Temporal Lobe Epilepsy.

Neuropsychological tests Left MTLE-HS	Groups		Level of significance		
	Pre-Op Mean (±S.D.)	Post-Op Mean (±S.D.)	Effect of Surgery	Effect of NCC	Surgery*NCC Interaction
Digit Span					
HS-ONLY	5.57 (2.33)	5.65 (2.29)			
HS-NCC	4.10 (1.63)	5.78 (3.40)	0.88	0.85	0.27
Inverted Digit Span					
HS-ONLY	4.51(1.75)	4.43 (2.11)			
HS-NCC	4.19(1.62)	4.26(1.82)	0.17	0.91	0.43
Boston Naming Test					
HS-ONLY	39.96 (10.33)	36.64 (09.83)			
HS-NCC	38.01 (11.13)	33.43 (11.09)	0.001*	0.39	0.21
Word Fluency (FAS)					
HS-ONLY	29.29 (8.47)	29.35 (8.65)			
HS-NCC	29.16 (8.82)	29.27 (8.33)	0.87	0.77	0.98
Logical Memory I					
HS-ONLY	16.51 (8.25)	14.28 (7.48)			
HS-NCC	16.16 (6.62)	12.46 (5.97)	0.003*	0.78	0.10
Logical Memory II					
HS-ONLY	10.00 (7.90)	8.83 (7.65)			
HS-NCC	08.96 (6.82)	7.35 (6.41)	0.04	0.60	0.71
Visual Reproduction I					
HS-ONLY	31.08 (6.79)	31.37 (6.35)			
HS-NCC	30.51 (5.76)	30.99 (6.55)	0.81	0.65	0.74
Visual Reproduction II					
HS-ONLY	20.35 (11.57)	20.03 (11.43)			
HS-NCC	20.90 (10.46)	21.67 (11.08)	0.53	0.10	0.57
RAVLT					
HS-ONLY	47.03 (10.61)	43.44 (10.89)			
HS-NCC	46.35 (09.75)	42.30 (10.68)	<0.001*	0.99	<0.90
RAVLT Post Interference					
HS-ONLY	8.39 (3.20)	7.28 (3.20)			
HS-NCC	8.12 (2.77)	7.10 (3.47)	0.002*	0.99	0.76
RAVLT 30 Minutes					
HS-ONLY	7.88 (3.65)	6.48 (3.45)			
HS-NCC	7.82 (3.52)	6.59 (3.79)	<0.001*	0.61	0.65
RAVLT rec					
HS-ONLY	12.82 (2.52)	12.44 (2.72)			
HS-NCC	12.88 (2.45)	12.32 (2.18)	0.32	0.60	0.47
RVDLT					
HS-ONLY	29.45 (12.22)	32.00 (14.79)			
HS-NCC	29.93 (14.55)	30.21 (14.62)	0.49	0.62	0.18
RVDLT Post Interference					
HS-ONLY	6.57 (3.44)	6.97 (3.85)			
HS-NCC	6.62 (3.40)	7.13 (3.60)	0.47	0.19	0.57
RVDLT 30 minutes					
HS-ONLY	6.14 (3.09)	6.78 (3.93)			
HS-NCC	6.51 (3.37)	6.96 (3.75)	0.96	0.86	0.86
RVDLT rec					
HS-ONLY	12.25 (2.16)	13.41 (2.58)			
HS-NCC	12.57 (1.94)	13.17 (1.62)	0.11	0.46	0.86
Rey Complex Figure Copy					
HS-ONLY	30.39 (6.14)	29.72 (6.25)			
HS-NCC	29.18 (6.94)	29.02 (7.50)	0.70	0.83	0.48
Rey Complex Figure 30 min					
HS-ONLY	11.49 (6.38)	12.37 (6.58)			
HS-NCC	11.19 (6.13)	11.96 (6.51)	0.93	0.92	0.90
QI					
HS-ONLY	81.59 (10.60)	82.64 9.76			
HS-NCC	78.82 (10.39)	79.93 9.84	0.09	0.32	0.96

(*) significant.

**Table 5:** Results of Pre- and Postsurgical Neuropsychological Performance for Right Mesial Temporal Lobe Epilepsy.

Neuropsychological tests Right MTLE-HS	Groups		Level of significance		
	Pre-Op Mean (±S.D.)	Post-Op Mean (±S.D.)	Effect of Surgery	Effect of NCC	Surgery*NCC Interaction
Digit Span					
HS-ONLY	5.60 (1.69)	5.45 (1.84)			
HS-NCC	5.84 (1.86)	5.82 (2.25)	0.46	0.35	0.63
Inverted Digit Span					
HS-ONLY	4.43(1.60)	4.71 (1.73)			
HS-NCC	4.63(1.92)	4.93(1.94)	0.07*	0.56	0.88
Boston Naming Test					
HS-ONLY	46.74 (09.53)	48.18 (09.36)			
HS-NCC	45.42 (10.47)	46.28 (09.77)	<0.001*	0.14	0.29
Word Fluency (FAS)					
HS-ONLY	29.22 (8.29)	31.32 (9.77)			
HS-NCC	32.36 (8.82)	33.04 (11.25)	0.92	0.18	0.23
Logical Memory I					
HS-ONLY	20.83 (7.99)	21.72 (8.28)			
HS-NCC	19.79 (8.73)	20.23 (8.63)	0.37	0.10	0.66
Logical Memory II					
HS-ONLY	14.81 (7.83)	17.08 (8.07)			
HS-NCC	14.30 (9.44)	14.79 (9.39)	0.15	0.08	0.09
Visual Reproduction I					
HS-ONLY	31.25 (5.89)	30.99 (7.17)			
HS-NCC	30.56 (6.23)	30.45 (6.92)	0.49	0.25	0.74
Visual Reproduction II					
HS-ONLY	21.45 (10.84)	21.60 (11.15)			
HS-NCC	20.07 (10.72)	19.67 (11.15)	0.82	0.15	0.70
RAVLT					
HS-ONLY	50.88 (10.23)	52.17 (10.69)			
HS-NCC	52.74 (11.63)	52.10 (11.23)	0.96	0.10	0.10
RAVLT Post Interference					
HS-ONLY	10.50 (2.76)	10.89 (2.93)			
HS-NCC	10.81 (3.20)	10.44 (3.48)	0.10	0.72	0.07
RAVLT 30 Minutes					
HS-ONLY	9.96 (3.04)	10.97 (2.72)			
HS-NCC	10.47 (3.61)	10.44 (3.45)	0.21	0.87	0.02
RAVLT rec					
HS-ONLY	13.78 (1.70)	14.01 (3.56)			
HS-NCC	13.79 (1.63)	13.71 (2.06)	0.70	0.67	0.62
RVDLT					
HS-ONLY	29.45 (12.22)	32.00 (14.79)			
HS-NCC	29.93 (14.55)	30.21 (14.62)	0.49	0.62	0.18
RVDLT Post Interference					
HS-ONLY	7.59 (3.88)	7.69 (3.63)			
HS-NCC	7.51 (3.81)	7.05 (3.39)	0.76	0.38	0.38
RVDLT 30 minutes					
HS-ONLY	7.08 (3.31)	7.26 (3.69)			
HS-NCC	7.21 (3.83)	6.79 (3.50)	0.45	0.57	0.21
RVDLT rec					
HS-ONLY	12.29 (2.51)	12.71 (2.21)			
HS-NCC	12.17 (2.09)	12.57 (2.40)	0.11	0.46	0.86
Rey Complex Figure Copy					
HS-ONLY	30.39 (6.14)	29.72 (6.25)			
HS-NCC	29.18 (6.94)	29.02 (7.50)	0.57	0.46	0.40
Rey Complex Figure 30 min					
HS-ONLY	11.21 (6.29)	11.70 (6.30)			
HS-NCC	10.43 (6.06)	10.26 (6.06)	0.64	0.09	0.48
QI					
HS-ONLY	84.54 10.19	86.29 10.58			
HS-NCC	85.45 11.45	85.30 11.74	0.47	0.73	0.04

(*) significant.

## Discussion

Overall, 126 (38.9%) of the 324 patients presented radiological findings compatible with cNCC. When considering neuropsychological data, our results showed that cNCC is not a risk factor for further cognitive decline after temporal lobectomy in patients with MTLE-HS plus cNCC. Ninety-four (74.6%) patients with MTLE-HS plus cNCC were Engel class I after surgery, rates that were not significantly different from those observed in patients with MTLE-HS alone (153 patients, 77.3%). However, the chances of patients achieving Engel class IA status were significantly lower in those with MTLE-HS plus cNCC when compared to patients with MTLE-HS alone (48.5% versus 31.7%). Also, patients with MTLE-HS plus cNCC showed higher rates of Engel class ID status after surgery. Therefore, regarding the presence of cNCC in MTLE-HS, we conclude that temporal lobectomy is effective for seizure control and is cognitively safe for most patients when video-EEG reveals seizure onset in the mesial temporal lobe. However, patients with MTLE-HS plus cNCC have reduced chances of achieving Engel class IA status after surgery, and are at higher risk of seizure recurrence if treatment is reduced or interrupted after surgery.

The effects of cysticercosis on the brain are still poorly understood. Some findings suggest that chronic forms of cNCC can present perilesional gliosis, a finding that has been associated with seizure, with surface EEG abnormalities and with other clinical abnormalities [31]-[33]. These results suggest that even a single calcified lesion might not be as silent as imagined, and therefore could influence cognitive performance or seizure control. Here we report that, although chances of postsurgical Engel class I status were similar in MTLE-HS patients with or without cNCC, the presence of cNCC was associated with lower chances of patients achieving Engel class IA status. Moreover, the presence of cNCC increases the chance of seizure recurrence with medication interruption. This is an interesting finding, and in line with the general view that surgical prognosis is different for patients with MTLE-HS plus another potentially epileptogenic lesion, the case of the dual pathology [34]–[37]. However, there are several subtypes of dual pathology combinations with different prognostic significance. Regarding cNCC associated with MTLE-HS, our finding that Engel class I outcomes are similar in MTLE-HS patients with or without cNCC, supports the notion that surgery is effective for controlling refractory seizures associated with HS. However, selected patients remain seizure-free only as long as they remain under pharmacological treatment. Attempts to reduce or interrupt medication in some MTLE-HS plus cNCC patients result in seizure recrudescence. Thus, Engel class ID outcomes were more commonly observed in MTLE-HS associated with cNCC than in MTLE-HS alone. As a conclusion, surgery is effective for controlling seizures in MTLE-HS plus cNCC, although drug discontinuation might result in seizure recurrence is selected patients. Unfortunately, we have few patients in this situation and this study was not designed to evaluate this specific question. A future study needs to be designed to specifically evaluate this interesting question.

NCC has been reported to be associated with impairment of several cognitive functions that varies according to the brain areas involved, the number of cysticerci, and the differences in the inflammatory response of each individual [16]–[21]. However, our results show that the cognitive effects of cNCC are not so evident in MTLE-HS patients, perhaps masked by the impairment that patients with MTLE-HS already have due to pathology, seizures, and drug therapy. Nevertheless, we conclude that cNCC was not a risk factor for further postsurgical cognitive decline after temporal lobectomy in MTLE-HS, one of the objectives of this study. This result further supports surgical decision-making in patients with MTLE-HS plus cNCC. Regarding cognition in dual pathology, our results differ from those observed by Bigel and Smith who showed that, in children, dual pathology is associated with lower cognitive performance when compared to patients who present HS alone [38]. However, in a previous study we have already observed that cNCC has little impact on neuropsychological performance in MTLE-HS patients [22]. In our view, these discrepancies could be due to the type, size, chronicity and pathophysiologic characteristics of the patient studied and to the area of the lesions associated with MTLE-HS. Further studies are necessary to investigate these aspects. On the other hand, our observations are in line with those of Martin et al. showing that dual pathology might not affect postsurgical cognitive prognosis in MTLE-HS patients [39]. Finally, if we consider results for Engel I (IA+IB+IC+ID) versus Engel II, III, and IV, our results for MTLE-HS plus cNCC patients further support that epilepsy surgery can remedy refractory symptomatic focal epilepsy (MTLE-HS) in patients with other coexisting more benign potentially epileptogenic focal lesions (cNCC) [40].

A closer look at our data suggests an additional interesting aspect. The prevalence of cNCC in our population has been estimated at 71.4/100,000 (based on recent unpublished epidemiological data obtained from the Public Health System of our region, an area where NCC notification is compulsory). However, some studies suggest that in specific regions the prevalence of NCC might be higher, although never exceeding 5% even in the most pessimistic estimates [29], [41]. In the best study available to us, Chimelli et al. observed that cNCC was detected in 1.5% of all 2522 autopsies done between 1992 and 1997 in our hospital [30]. Because that study was conducted at our hospital, it evaluated a large cohort, and was based on the population living in our region, we consider this estimate to be as close as possible to the real prevalence of cNCC in our area. An intriguing result of the present study was the occurrence of radiological findings of cNCC in 37.2% of our patients, an incidence indeed far higher than expected. Moreover, there was an unexpected statistically significant difference in cNCC findings regarding sex. Even though cNCC equally affects both genders or seems to be slightly more common in the male population of Brazilian patients, women develop more severe forms of cNCC [29], [41], [42]. The high prevalence of cNCC in patients with MTLE-HS, taken together with the observation that cNCC is more commonly observed in female patients, may suggest that cNCC might be, per se, a possible cause of MTLE-HS, as previously observed by us and by others [43], [44]. Our clinical observation suggesting that NCC might be a cause of MTLE-HS in some patients is interesting and might contribute to a growing discussion about whether or not NCC contributes to the development of MTLE-HS or might even cause it in some patients.

Our study has some limitations that should be acknowledged. It is retrospective and conducted only at one center. Moreover, these results might not be valid for those rare patients who present more severe forms of cNCC or those rare patients that might experience refractory seizure due to cNCC [26]. In spite of these limitations, ours is the first study showing that temporal lobectomy is cognitively safe in MTLE-HS plus cNCC and the first cohort study analyzing the surgical outcome of seizures when cNNC is observed in association with MTLE-HS. Moreover, because of the epidemiological design used here, the large cohort studied, and the appropriate follow-up period, we believe that our observations have intrinsic as well as extrinsic validity and thus could be appropriately extrapolated to other patients similar to those studied here.

In conclusion, we observed that cNCC frequently occurs in association with MTLE-HS in endemic zones, perhaps suggesting a cause-effect relationship and an association that might affect a large number of patients worldwide [45], [46]. In spite of some differences for Engel class IA or ID outcome, our data support that temporal lobectomy is effective for long-term seizure control in patients with MTLS-HS plus cNCC when MRI is compatible with MTLE-HS and a video-EEG documents seizure onset in the mesial temporal lobe. Moreover, the procedure is cognitively safe for most patients affected by MTLS-HS plus cNCC. As a whole, our study provides consistent evidence supporting the surgical decision for seizure treatment in patients with MTLE-HS who also present neuroradiological findings of cNCC. Because the association of MTLE-HS and cNCC might be commonly observed in several world regions, we believe this study could be relevant for therapeutic decisions in a large number of patients worldwide.
